# A Case Report on Protease Serine 1 (PRSS1)-Related Acute Pancreatitis

**DOI:** 10.7759/cureus.80616

**Published:** 2025-03-15

**Authors:** Vimalraj Vijayakumar, Premkumar K, Ratnakar Kini P

**Affiliations:** 1 Department of Medical Gastroenterology, Madras Medical College, Chennai, IND; 2 Department of Gastroenterology, Madras Medical College, Chennai, IND

**Keywords:** acute pancreatitis (ap), genetic panel testing, hereditary pancreatitis, prss1 mutation, ugt1a1 gene

## Abstract

Acute pancreatitis (AP) is a multifactorial disease. Genetic predisposition as the etiology of AP is rare. We report the case of a 29-year-old male patient who presented with symptoms suggestive of AP. A complete workup, including genetic analysis, revealed the presence of both protease serine 1 (PRSS1) and UDP Glucuronosyltransferase family 1 member A1 (UGT1A1) mutations. Both PRSS1 and UGT1A1 mutations can cause AP by different mechanisms. This case has been reported because of the novelty of the two different genetic mutations in the same individual that could independently increase pancreatitis risk. The possible synergistic effect has not been reported previously. Understanding this interaction emphasizes the importance of genetic testing in AP.

## Introduction

Acute pancreatitis (AP) is an inflammatory disease of the pancreatic tissue. The global incidence of AP varies from 30 to 40 cases per lakh. The actual burden of the disease on the Indian subcontinent remains unknown. The AP Patient Registry to Examine Novel Therapies in Clinical Experience is a large prospectively collected registry that quotes alcohol (44.5%), followed by biliary (27.9%), a common cause of AP in India [1]. The AD mutation in protease serine 1 (PRSS1), which encodes human cationic trypsinogen, the precursor of the most abundant digestive enzyme secreted by the pancreas, was the first genetic abnormality detected in AP. Various other pancreatitis-related risk genes, including SPINK1, CFTR, CLDN2, CASR, CPA1, CEL, CTSB, MYO9B, and UBR1, have been identified [2]. The prevalence of the mutation in the UDP Glucuronosyltransferase family 1 member A1 (UGT1A1) gene has been reported to be 30%-45% in the Indian population [3]. Reduced UGT1A1 activity leads to indirect hyperbilirubinemia called Gilbert syndrome (GS), which increases the risk of gallstones and oxidative stress, predisposing to AP [4]. PRSS1 mutations have been proven to cause AP, whereas direct causation of AP due to UGT1A1 mutations has not been reported. Combined PRSS1 and UGT1A1 mutation has not been reported in the literature so far. This case is unique because it demonstrates the rare coexistence of two genetic mutations, PRSS1 and UGT1A1, and their potential in causing AP in the same patient.

## Case presentation

**Figure 1:**
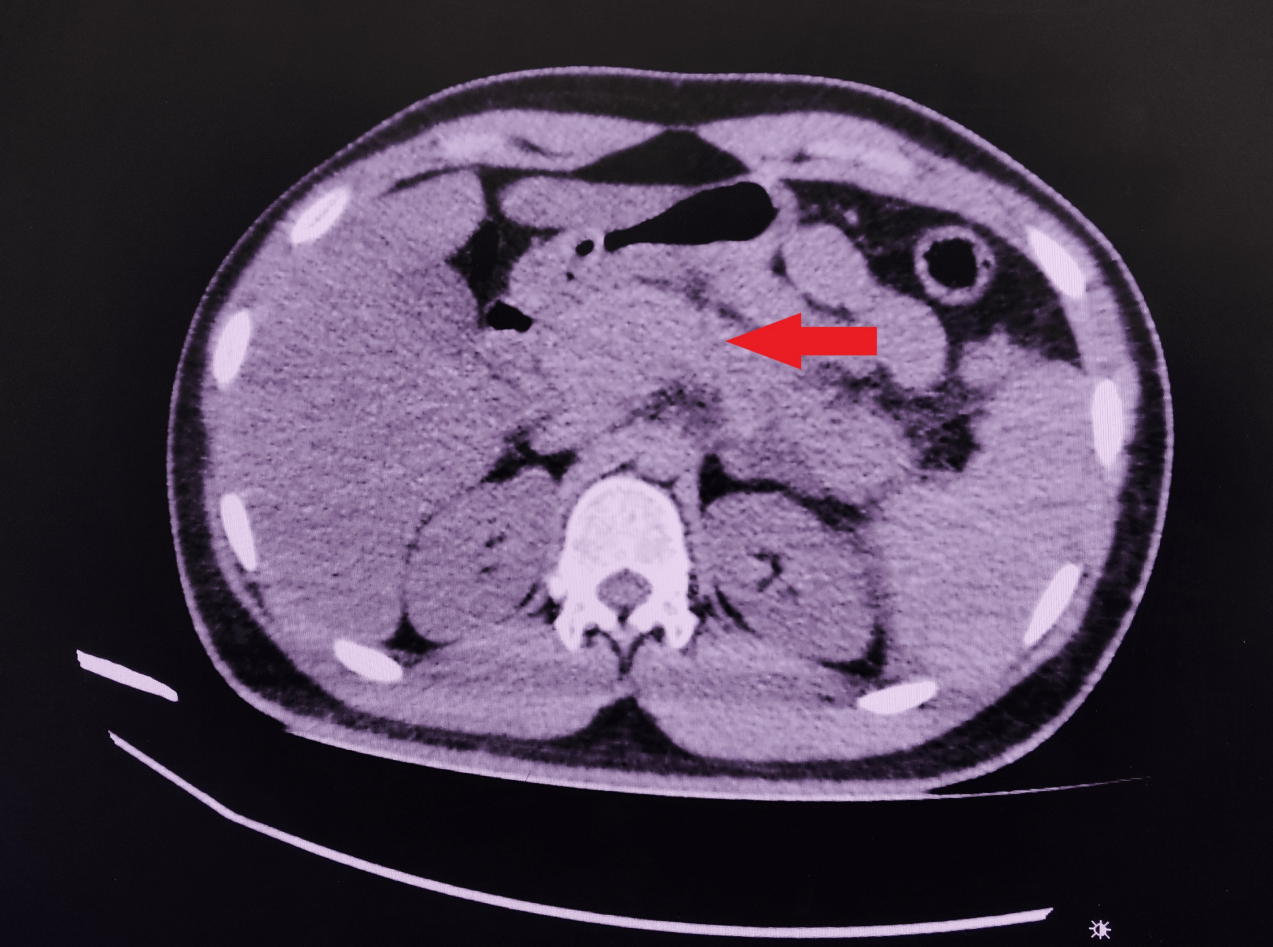
CT scan axial cut showing bulky pancreas with peripancreatic fat stranding (red arrow) CT: computed tomography

A 29-year-old male patient presented with complaints of abdominal pain for four days. The pain was confined to the epigastric region with severe intensity, radiating to the back, and aggravated by food intake. Pain was associated with vomiting and nonbiliousness and improved with medication. There were no fever, jaundice, or bleeding symptoms, and his bowel habits were normal. The patient denied any past episodes of abdominal pain or jaundice. He also denied the use of drugs, smoking, medications, or alcohol consumption and did not undergo any abdominal surgery. None of the family members had similar complaints, and there was no chronic illness in his family. Examination revealed epigastric tenderness, and the rest of the abdomen and other systemic examinations were within normal limits. Blood investigations, including complete hemogram and renal and liver function tests, were normal. His serum amylase and lipase levels were 591 international units (IU)/L (<40 IU/L) and 728 IU/L (<40 IU/L), respectively. The patient was provisionally diagnosed with AP. Further blood workup including calcium was 9.5 mg/dL (9-11 mg/dL), and serum triglyceride was 134 mg/dL (<150 mg/dL). A computed tomography (CT) scan reported a diffuse parenchymal enlargement with peripancreatic fat stranding without fluid collection and necrosis, suggesting AP (Figure 1). The gallbladder was normal without gallstones. The antinuclear antibody test results were negative. Since most of the causes of AP were ruled out, to clarify the etiology of AP, genetic testing was done, which found to have heterozygous missense pathogenic variant in exon 2 of the PRSS1 gene (c.86A>T, p.N29I) and a homozygous pathogenic variant in the promoter region of the UGT1A1 gene, i.e., c.-40_-39 insTA or (TA)₇. The patient was managed conservatively, and his symptoms improved. His family members were advised to undergo genetic testing for the PRSS1 mutation, but this was not performed due to financial constraints. The patient was informed about recurrent pancreatitis episodes and the risk of chronic pancreatitis and subsequent malignancy and was advised to follow up regularly.

## Discussion

Pancreatitis is a heterogeneous disease triggered by multiple factors, such as environmental (alcohol), metabolic, and anatomical factors, including ductal anomalies, obstructive diseases, and autoimmune diseases [5]. The exact etiology of AP is unknown in approximately 10% of cases. Although the first case of hereditary pancreatitis (HP) was reported by Steinberg and Comfort in 1952, the PRSS1 gene was discovered as the gene responsible for Whitcomb 44 years later in 1996 [6]. Since then, multiple genes implicated in the pathogenesis of AP have been identified (Table 1). Excluding PRSS1, other genes act as predisposing factors that are associated with environmental and metabolic factors, such as alcohol and hyperlipidemia, lowering the threshold for pancreatitis [7]. PRSS1 is located on chromosome 7q35 and encodes cationic trypsinogen, which activates trypsin and aids in protein digestion under normal conditions. Increased trypsinogen autoactivation initiating autodigestion and inflammation is the primary mechanism by which gain-of-function mutations in PRSS1 cause pancreatitis. Reduced trypsin degradation, altered calcium homeostasis, and reduced trypsin inhibitory control are other possible mechanisms [8]. HP caused by a PRSS1 mutation is inherited in an AD manner, with incomplete penetrance. However, the proportion of PRSS1-related pancreatitis cases caused by de novo pathogenic variants remains unknown. In our case, none of the family members were clinically affected, and they were not tested for PRSS1 mutations due to financial constraints. In cases of negative familial association, a possible explanation could be a de novo variant or parental germline mosaicism [9]. Notable pathogenic variants such as p.R122H and p.N29I account for 90% of the cases. Some less common variants were p.A16V, p.D21A, p.K23R, p.R116C, and p.V39A (Table 2). Most of these variants have been reported in single families. Patients with PRSS1-related HP have a 40%-55% lifetime risk of pancreatic malignancy. Variants such as p.L104V have been reported in families with solid pseudopapillary tumors of the pancreas in China [10]. In a previous case report, loss-of-function PRSS1 variant p.Y37X was found in chronic alcoholics without pancreatitis. This variant has been suggested to protect against chronic pancreatitis. Genetic analysis in our patient revealed homozygous polymorphism in the UGT1A1 gene characterized by the insertion of additional dinucleotide sequence (TA) in the promoter region, A(TA)₇TAA, suggestive of GS. There are no data related to the prevalence of pancreatitis in patients with GS. Patients with GS have elevated levels of monoconjugated bile pigments, which increase the risk of pigmented gallstones, which may lead to biliary pancreatitis [11]. This may explain the significance of the UGT1A1 promoter region mutation in this case. It has also been described in the literature that not all patients with promoter region mutations will have GS, as in our case, where bilirubin levels were normal. Our patient with AP without a family history was found to have both PRSS1 and UGT1A1 promoter region mutations. The potential interaction between PRSS1 and UGT1A1 mutation acting synergistically or independently in the pathogenesis of AP has not been reported previously. In this case, since there was no obvious gallstone or sludge demonstrated on imaging, the etiology of AP is primarily attributed to the PRSS1 gene mutation. Family members have to be screened for PRSS1 and UGT1A1 mutations, which was not done in this case. Long-term follow-up of PRSS1-related AP is advised because of the risk of chronic pancreatitis and pancreatic cancer in the future.

**Table 1:** List of prominent gene mutated in pancreatitis AP: acute pancreatitis; PRSS: protease serine

Gene	Inheritance	Clinical relevance
PRSS	Autosomal dominant	AP/recurrent AP, chronic pancreatitis
SPINK1	Autosomal recessive	Early onset disease, aggressive disease
CFTR	Autosomal recessive	Cystic fibrosis, chronic pancreatitis
CASR	Autosomal dominant	Hypercalcemia AP/recurrent AP, chronic pancreatitis
CLDN2	X linked	Alcohol-related pancreatitis
CEL	Autosomal dominant	Diabetes mellitus, pancreatic lipomatosis, chronic pancreatitis
CCPA1	Autosomal dominant	Early onset disease, chronic pancreatitis
CTRC	Autosomal dominant	AP/recurrent AP, chronic pancreatitis

**Table 2:** List of important pathogenic PRSS1 gene variants PRSSI: protease serine 1

Variant	Nucleotide change	Mechanism
p.R122H	c.365G>A	Increased autoactivation
p.N29I	c.86A>T	Increased autoactivation
p.D19A	c.56A>C	Increased autoactivation
p.K23R	c.68A>G	Increased autoactivation
p.K92N	c.276G>T	Misfolding of proteins
p.C139S	c.415T>A	Misfolding of proteins
p.R122C	c.364C>T	Increased autoactivation
p.A16V	c.47C>T	Increased autoactivation
p.L104P	c.311T>C	Misfolding of proteins
p.D22G	c.65A>G	Increased autoactivation
p.D21A	c.62A>C	Increased autoactivation
p.V39A	c.116T>C	Increased autoactivation
p.D100H	c.298G>C	Misfolding of proteins
p.R116C	c.346C>T	Misfolding of proteins
p.p.S124F	c.371C>T	Misfolding of proteins

## Conclusions

In conclusion, this case report highlights that genetic testing should be performed in patients with unexplained AP. PRSS1 variant-related pancreatitis can present de novo without affecting family members. Coexistence of PRSS1 and UGT1A1 mutations is rare and has not been reported previously in literature. Considering the risk of pancreatic malignancy, genetic counseling and regular follow-up of these patients should be considered.
